# Überregionale Public-Health-Akteure in Deutschland – eine Bestandsaufnahme und Kategorisierung

**DOI:** 10.1007/s00103-021-03456-0

**Published:** 2021-12-03

**Authors:** Franziska Hommes, Amir Mohsenpour, Dana Kropff, Lisa Pilgram, Svenja Matusall, Peter von Philipsborn, Kerstin Sell

**Affiliations:** 1 Nachwuchsnetzwerk Öffentliche Gesundheit, Berlin, Deutschland; 2 Charité – Universitätsmedizin Berlin, Institut für Tropenmedizin und Internationale Gesundheit, Berlin, Deutschland; 3 AG Bevölkerungsmedizin und Versorgungsforschung, Fakultät für Gesundheitswissenschaften, Universität Bielefeld, Universitätsstraße 25, 33615 Bielefeld, Deutschland; 4 Institut für Afrikastudien, Universität Leipzig, Leipzig, Deutschland; 5 Abteilung für Innere Medizin, Hämatologie/Onkologie, Goethe-Universität Frankfurt, Frankfurt am Main, Deutschland; 6 Zukunftsforum Public Health, Berlin, Deutschland; 7 Pettenkofer School of Public Health, München, Deutschland; 8 Lehrstuhl für Public Health und Versorgungsforschung, Institut für medizinische Informationsverarbeitung, Biometrie und Epidemiologie – IBE, LMU München, München, Deutschland

**Keywords:** Gesundheitsfachkräfte, Öffentliche Gesundheit, Berufswege, Nachwuchsfachkräfte, Public-Health-System, Public health, Public health systems, Career paths, Young professionals, Health workforce

## Abstract

**Hintergrund:**

Akteure der öffentlichen Gesundheit (Public Health) tragen wesentlich zu Gesundheitsschutz, -förderung und Prävention auf Bevölkerungsebene bei. Kenntnisse über die Akteurslandschaft erleichtern die Zusammenarbeit und erhöhen die Transparenz des Berufsfeldes für Nachwuchsfachkräfte.

**Zielsetzung:**

Ziel dieser Arbeit waren eine Bestandsaufnahme und Kategorisierung überregionaler Public-Health-Akteure in Deutschland.

**Methodik:**

Ausgehend von einer Aufstellung der teilnehmenden Institutionen am Zukunftsforum Public Health und zielgerichteter Onlinerecherche wurden überregionale Organisationen mit Public-Health-Schwerpunkt identifiziert. Alle Akteure wurden durch ≥ 2 Autor:innen unabhängig voneinander gescreent. Rein lokal tätige Akteure und solche ohne erkennbaren Public-Health-Schwerpunkt wurden ausgeschlossen. Mittels Schneeballverfahrens wurden weitere Akteure identifiziert. Zur thematischen Clusterung wurde induktiv ein Kategoriensystem gebildet.

**Ergebnisse:**

Von 645 gescreenten Akteuren wurden 307 (47,6 %) eingeschlossen und 12 Ober- sowie 30 Subkategorien zugeordnet. Die Oberkategorie Fachverbände (*n* = 60) weist die höchste Akteursanzahl auf, gefolgt von zivilgesellschaftlichen (*n* = 49) und staatlichen Akteuren (*n* = 40). Neben einer tabellarischen und grafischen Darstellung wurde eine interaktive Akteursübersicht erstellt (www.noeg.org).

**Diskussion:**

Diese Arbeit bietet eine umfangreiche Übersicht über Akteure der öffentlichen Gesundheit in Deutschland und verdeutlicht die Breite der deutschen Public-Health-Landschaft. Die Ergebnisse bieten neue Möglichkeiten zur Vernetzung und können Nachwuchsfachkräften Berufswege aufzeigen. Ausgehend von dieser Arbeit sind ergänzende Forschungsarbeiten zu Public-Health-Akteuren sinnvoll.

**Zusatzmaterial online:**

Zusätzliche Informationen sind in der Online-Version dieses Artikels (10.1007/s00103-021-03456-0) enthalten.

## Abstract (en)

**Background:**

Public health actors contribute significantly to health protection, promotion, and prevention at the population level. An overview of the public health landscape can facilitate collaboration among the stakeholders and increase transparency of career paths for young professionals.

**Objectives:**

This study aims to develop an overview and category system of supra-regional public health actors in Germany.

**Methods:**

Based on a list of institutional actors that participated in the national conference Future Forum Public Health (“Zukunftsforum Public Health”) and a targeted online search by the authors, supra-regional institutions and organizations with a public health focus were identified. All actors were screened independently by ≥ 2 authors. Community-level actors as well as those without a direct public health focus were excluded. Additional actors were identified via a snowballing process. In order to cluster the actors thematically, a category system was formed inductively.

**Results:**

Out of a total of 645 screened actors, 307 (47.6%) were included and subsequently assigned to 12 main and 30 subcategories. Professional associations (*n* = 60) made up the largest category, followed by civil society (*n* = 49) and state actors (*n* = 40). In addition to a tabular and graphical overview, an interactive visualization of the actors was created (www.noeg.org).

**Conclusions:**

This study provides a comprehensive overview of supra-regional institutional public health actors in Germany and highlights the breadth of the German public health landscape. The results of this work offer new opportunities for collaboration and can support young professionals in their career paths. Building on this work, further research on public health actors is recommended.

## Hintergrund

Akteure der öffentlichen Gesundheit leisten einen wesentlichen Beitrag zu Gesundheitsschutz, Gesundheitsförderung und Prävention auf Bevölkerungsebene. Neben dem Öffentlichen Gesundheitsdienst (ÖGD) existiert in Deutschland eine Vielzahl an Organisationen und Institutionen, deren Ziel es ist, „durch organisierte gesellschaftliche Anstrengungen Krankheit zu vermeiden, Leben zu verlängern und Gesundheit zu fördern“, und die damit als Public-Health-Akteure gelten können [1]. Diese Akteure arbeiten von der lokalen bis zur internationalen Ebene in verschiedensten Praxis‑, Wissenschafts- und Politikbereichen. In Deutschland galt die Public-Health-Landschaft lange Zeit als fragmentiert [2, 3]. Seit mehreren Jahren bestehen jedoch in der Public-Health-Gemeinschaft Bestrebungen, dieser Zersplitterung entgegenzuwirken und weitere Reformen im Bereich der öffentlichen Gesundheit zu initiieren [4, 5]. Diese Reformprozesse wurden unter anderem durch ein Gutachten der Nationalen Akademie der Wissenschaften Leopoldina von 2015 angestoßen [6] und haben durch die SARS-CoV-2-Pandemie neue Dynamik und öffentliche Aufmerksamkeit erfahren [7–9].

Von politischer Seite ist mit dem Pakt für den Öffentlichen Gesundheitsdienst eine umfangreiche Stärkung des ÖGD initiiert worden, die insbesondere auf die Bekämpfung pandemischer Notlagen ausgerichtet ist [10]. Erforderlich ist darüber hinaus auch eine langfristige, umfangreiche Stärkung öffentlicher Gesundheit in Deutschland [6, 11]. Seit mehreren Jahren besteht mit dem Zukunftsforum Public Health (ZfPH) eine breit aufgestellte Reforminitiative, in der sich Institutionen und Individuen aus Wissenschaft und Praxis für die Erarbeitung einer visionären Public-Health-Strategie für Deutschland zusammengeschlossen haben. Im Rahmen der seit 2016 stattfindenden Symposien kam eine Vielzahl an Akteuren zusammen, die verschiedene Themenschwerpunkte, Interessen und Hintergründe der öffentlichen Gesundheit repräsentieren [12]. Das 2017 von einigen der Autor:innen mitgegründete Nachwuchsnetzwerk Öffentliche Gesundheit (NÖG) zielt darauf ab, Austausch und Vernetzung zwischen Studierenden, Nachwuchskräften und an Nachwuchsförderung Interessierten im Bereich Public Health zu fördern. Das NÖG engagiert sich unter anderem für Reformen der Aus‑, Fort- und Weiterbildung in der öffentlichen Gesundheit, insbesondere mit dem Ziel, die Interdisziplinarität des Fachs zu stärken und Berufswege transparenter zu machen. Die vorliegende Arbeit soll in diesem Zusammenhang zentrale Herausforderungen für Public-Health-Nachwuchsfachkräfte in Deutschland adressieren: Die mit der Vielfalt von Public Health einhergehende Unübersichtlichkeit des Berufsfelds und die daraus resultierende Intransparenz vieler Berufswege [13].

Zwar existieren bereits Darstellungen der Struktur des ÖGD [14] sowie des Gesundheitssystems [15] in Deutschland, diese bilden jeweils jedoch nur Teilaspekte und nicht die aktuelle Vielfalt von Public Health in Deutschland ab. Der ÖGD stellt mit den staatlichen Institutionen der Gesundheitsverwaltung auf Bundes‑, Länder- und kommunaler Ebene einen Teil des Public-Health-Systems mit umfassenden Aufgaben, unter anderem im Bereich Gesundheitsschutz, Gesundheitsförderung und Gesundheitsberichterstattung, dar. Diese Aufgaben des ÖGD – vom aktuell im Fokus stehenden Infektionsschutz bis zum sozialkompensatorischen, planerischen und gesundheitsförderlichen Engagement insbesondere in kommunalen Settings – gehen mit der Zusammenarbeit einer Vielzahl weiterer Partnerorganisationen und Akteure einher [16]. Die Public-Health-Akteure außerhalb des ÖGD tragen so zu einem weit gefassten, vernetzten Public-Health-System bei.

Kenntnisse zu beteiligten Akteuren in Deutschland im Public-Health-Bereich spielen damit nicht nur für Nachwuchsfachkräfte in der beruflichen Orientierung eine wichtige Rolle, sondern dienen auch der weiteren Vernetzung und Koordination der Public-Health-Akteure untereinander. Letzteres ist neben einer Koordination und Kooperation im Bereich der Forschung insbesondere vor dem Hintergrund aktueller Reformprozesse in der öffentlichen Gesundheit relevant. Bislang existiert unserem Wissen nach keine solche Kartierung der Public-Health-Landschaft in Deutschland.

Ziel dieser Arbeit ist es, durch die Identifikation, systematische Kategorisierung und grafische Darstellung institutioneller Public-Health-Akteure in Deutschland die bestehende Lücke einer überregionalen Public-Health-Akteursübersicht zu schließen.

## Methodik

Die Datenerhebung und -auswertung wurde von Mitgliedern der AG Berufswege des NÖG zwischen September 2020 und Februar 2021 durchgeführt.

### Identifikation von Public-Health-Akteuren

Public-Health-Akteure wurden im Rahmen dieses Projekts als Organisationen und Institutionen definiert, die überregional oder deutschlandweit organisiert sind und die Public Health oder einzelne Gesundheitsthemen auf Bevölkerungsebene in mindestens einem ihrer Arbeitsbereiche aufgeführt haben. Zur Identifikation dieser Akteure wurden zunächst die folgenden Quellen genutzt: i) eine Liste aller teilnehmenden Akteure der ZfPH-Symposien 2016–2020, ii) eine Akteurssammlung, die 2017–2018 durch die Geschäftsstelle des ZfPH anhand nicht systematischer Recherchen erstellt wurde, und iii) eine zielgerichtete Onlinerecherche in PubMed und Google (auf Basis der Stichwörter *Public Health* oder *Öffentliche Gesundheit* oder *Öffentliches Gesundheitswesen* und *Deutschland* oder *Germany*).

Zur Ergänzung dieser Quellen wurde ein selektives Schneeballverfahren durchgeführt. Hierfür wurden solche Akteure ausgewählt, bei denen aufgrund ihrer Charakteristika von einer großen Wahrscheinlichkeit der Identifikation weiterer relevanter Akteure ausgegangen werden konnte. Zu diesen Charakteristika gehörten insbesondere zivilgesellschaftliche Netzwerke und institutionelle Arbeitsgemeinschaften. Durch ein Screening der Mitglieds‑, Partner- und/oder Förderorganisationen dieser Akteure auf ihren jeweiligen Homepages wurden weitere Akteure identifiziert.

### Screening

Nach einer gemeinsamen Diskussion über das eigene Public-Health-Verständnis und einer Verständigung der für das Screening verantwortlichen Autor:innen auf die Definition nach Acheson [1] als theoretischen Hintergrund wurde für die Operationalisierung der Fragestellung folgende Arbeitsdefinition entwickelt: Als Public-Health-Akteur wurden Institutionen und Organisationen definiert, die mindestens eine Aktivität oder Tätigkeit im Bereich Gesundheit mit klar erkennbarem Bevölkerungsbezug aufwiesen. Damit grenzen sich diese Akteure von primär individualmedizinischen Institutionen im Gesundheitsbereich ab sowie von solchen Akteuren, die zwar Relevanz für die öffentliche Gesundheit aufweisen, Gesundheit auf Bevölkerungsebene jedoch nicht als eigenen Schwerpunkt beschrieben haben (Health-in-all-Policies [HiAP]).

Alle Akteure wurden anhand der auf ihrer Homepage veröffentlichten Informationen (z. B. Arbeitsschwerpunkte, Leitlinien oder Satzungen) sowie bei Bedarf anhand ergänzender Sekundärquellen auf das Vorhandensein einer Tätigkeit im Bereich Gesundheit mit Bevölkerungsbezug gescreent. Dabei wurden die Akteure mithilfe der Software Rayyan (Qatar Computing Research Institute, HBKU, Doha, Qatar) durch jeweils ≥ 2 der Autor:innen (FH, AM, DK, LP) unabhängig voneinander gesichtet und eine Bewertung über Ein- oder Ausschluss vorgenommen [17]. Abweichungen in der Bewertung wurden im Forschungsteam diskutiert, bis ein Konsens erreicht wurde. Nach der Identifizierung der thematischen Arbeitsschwerpunkte wurden diese zu übergeordneten Themenbereichen zusammengefasst (z. B. Gesundheit und Umwelt/Klimawandel, Suchtprävention, Gesundheit von Kindern und Jugendlichen).

Um einer breiten Zielgruppe einen Überblick über die deutschlandweite Public-Health-Landschaft bieten zu können, lag der Fokus dieser Arbeit auf überregionalen Akteuren. Somit wurden folgende Ausschlusskriterien definiert (Tab. 1):

- rein auf lokaler oder kommunaler Ebene tätige Akteure;
- Untergruppen überregionaler Akteure;
- Akteure, die zwar mit ihrer Arbeit und/oder den Auswirkungen ihrer Aktivitäten die öffentliche Gesundheit in Deutschland beeinflussen, ohne dabei aber öffentliche Gesundheit als genuinen Arbeitsschwerpunkt zu haben;
- Akteure mit Global-Health-Schwerpunkt (s. nachfolgende Definition).

**Table 1:** 

Ausschlussgrund	Beschreibung	Anzahl Akteure
Untergruppe	Alle Akteure, die einer übergeordneten Struktur zugeordnet werden konnten (z. B. lokale Ableger eines überregionalen Vereins; Landesstruktur eines bundesweit tätigen Sozialversicherungsträgers). Bei Eignung wurde die jeweils höher gelegene institutionelle Ebene des Akteurs aufgenommen	58
Nicht erkennbarer Public-Health-Arbeitsschwerpunkt	Akteure, bei denen kein expliziter Public-Health-Arbeitsschwerpunkt erkennbar war. Die jeweilige Homepage des Akteurs, Organigramme, Satzungen, Leitbilder und ggf. Sekundärquellen dienten als Entscheidungsgrundlage	43
Lokaler Akteur	Akteure, welche in einem geografisch lokal begrenzten Raum agieren (z. B. Fachschaftsprojekte, städtische Initiativen mit Begrenzung auf eine Universität bzw. Stadt)	37
Kommunale Behörde	Staatliche Akteure, die auf kommunaler Ebene agieren (z. B. Gesundheits- und Veterinärämter)	32
Global Health	Akteure mit Fokus auf Themen der globalen Gesundheit, ohne direkten Bezug zur öffentlichen Gesundheit in Deutschland	15
Nicht auffindbar	Akteure mit nicht auffindbarer oder inaktiver Homepage	8

Zu lokalen bzw. kommunalen Akteuren zählten beispielsweise studentische Initiativen an einzelnen Universitäten, ehrenamtliche Arbeitsgruppen ohne überregionalen Netzwerkcharakter, kommunale Gesundheitsämter und Veterinärämter sowie kommunale medizinische Versorgungseinrichtungen. In Bezug auf Strukturen des ÖGD wurden alle identifizierten Einrichtungen auf Landes- und Bundesebene eingeschlossen. Bei Untergruppen handelte es sich um lokale Vertretungen überregionaler Institutionen (z. B. IPPNW Frankfurt), Teilbereiche einer übergeordneten Institution (z. B. Initiative für gesunde Ernährung IN FORM) oder akademische Institute einer Hochschule mit Lehre und Forschung zu Public Health (z. B. Mannheimer Institut für Public Health, Sozial- und Präventivmedizin). Hier wurde die jeweils strukturell übergeordnete Institution eingeschlossen, wenn die Einschlusskriterien erfüllt waren (z. B. Deutsche Sektion der IPPNW; Bundesanstalt für Landwirtschaft und Ernährung; Universität Heidelberg).

Strukturell und inhaltlich ähnliche Akteure, die beispielsweise durch die föderale Struktur Deutschlands mehrfach als Akteure identifiziert wurden, wie etwa Landesärztekammern oder Mitgliedsgesellschaften der Arbeitsgemeinschaft der wissenschaftlichen medizinischen Fachgesellschaften (AWMF) wie die Deutsche Gesellschaft für Sozialmedizin und Prävention e. V. (DGSMP) wurden aus Gründen der Übersichtlichkeit zu jeweils einem aggregierten Akteur zusammengefasst. Aufgrund der Vielzahl an Hochschulen mit Public-Health-Schwerpunkt wurde auch hier eine Aggregierung vorgenommen; für eine individuelle Darstellung verweisen wir auf existierende Verzeichnisse wie eine Übersicht zu Public-Health-Studiengängen [18].

Als Global-Health-Akteure wurden in Abgrenzung zu Public-Health-Akteuren jene definiert, die sich primär supraterritorialen Gesundheitsherausforderungen widmen, die ohne eine globale Kooperation nicht bewältigt werden können. Hierzu gehören grenzüberschreitende Gesundheitsfragen, welche Nationalstaaten nicht im Alleingang bewältigen können, wie die Auswirkungen des Klimawandels, zunehmende Antibiotikaresistenzen oder pandemische Notlagen [19]. Außerdem wird mit Global Health oft das normative Ziel einer globalen gesundheitlichen Chancengerechtigkeit verbunden [20]. Für eine Übersicht von Global-Health-Akteuren verweisen wir auf einschlägige Veröffentlichungen [21]. Der Hauptfokus dieser Arbeit liegt auf Public-Health-Akteuren* in* Deutschland. Internationale Akteure wie EU-Institutionen haben jedoch auch einen großen Einfluss auf Public Health in Deutschland, sodass diese Akteure trotz ihrer Position außerhalb Deutschlands in die Oberkategorie „Internationale Akteure“ aufgenommen wurden.

### Bildung des Kategoriensystems

Alle eingeschlossenen Akteure wurden thematisch geclustert, um induktiv Ober- und Subkategorien zu bilden. Dabei wurden die Akteure derjenigen Kategorie zugeordnet, die nach Konsens der Autor:innen am zutreffendsten die Zugehörigkeit abbildete. Die Ober- und Subkategorien, ihre Beschreibungen und die jeweilige Anzahl an Akteuren sind in Tab. 2 zusammengefasst. Die Granularität der Subkategorien richtete sich nach der Anzahl und Vielseitigkeit der eingeschlossenen Akteure.

**Table 2:** 

Oberkategorien *Subkategorien*	Beschreibung	Anzahl
**Fachverbände**	Akteure, die einen Zusammenschluss von mehreren Mitgliedern – meist Institutionen und ggf. zusätzlichen Einzelpersonen – bilden und die zu einem umschriebenen fachlichen Gesundheitsthema arbeiten. Oftmals geht die Zuordnung der Akteure zu dieser Oberkategorie auf die Selbstbezeichnung der Akteure zurück. Fachverbände sind abzugrenzen von rein zivilgesellschaftlichen Akteuren, von Berufsverbänden (berufsständische Interessensvertretung von Mitgliedern eines Berufsstandes) und von wissenschaftlichen Fachgesellschaften (Fokus auf die wissenschaftliche Arbeit, bspw. bei der Erstellung von Leitlinien durch wissenschaftlich-medizinische Fachgesellschaften)	**60**
*Arbeitsgemeinschaften*	Akteure, bei denen verschiedene Einzelpersonen und/oder Organisationen zu einem definierten fachlichen Gesundheitsthemengebiet zusammenarbeiten	41
*Dachverbände*	Akteure, die mehrere ähnlich strukturierte Organisationen zur fachlichen Zusammenarbeit vereinen und/oder sich selbst als Dachverband bezeichnen. Oftmals vertritt der Dachverband die Interessen der Mitgliedsorganisationen auf Bundesebene	19
**Zivilgesellschaftliche Akteure**	Zivilgesellschaftliche Akteure, z. B. Zusammenschlüsse von Individuen oder mehreren zivilgesellschaftlich organisierten Verbänden mit Public-Health-Arbeitsschwerpunkt, die nicht eindeutig anderen Oberkategorien zuzuordnen waren (z. B. Stiftungen, Fachverbände)	**49**
*Wohlfahrtsverbände*	Akteure der freien Wohlfahrtspflege, welche deutschlandweit soziale Unterstützung leisten	8
*Patient:innenverbände und Selbsthilfeorganisationen*	Organisationen, die zivilgesellschaftlich organisiert die Interessen von Patient:innen vertreten oder der Selbsthilfe dienen. Oftmals sind Patient:innen oder Betroffene selbst Mitglied/Leitung dieser Organisationen. Eine Mitgliedschaft ist i. d. R. unabhängig vom professionellen Hintergrund	5
*Studierenden- und Nachwuchsorganisationen*	Organisationen, die von Studierenden oder Nachwuchskräften gegründet/initiiert wurden und deren Mitglieder vorrangig Studierende und/oder Nachwuchskräfte sind	5
*Weitere Non-Profit-Organisationen*	Akteure der Zivilgesellschaft mit Public-Health-Arbeitsschwerpunkt, die keiner anderen Subkategorie zuzuordnen waren	31
**Staatliche Akteure**	Staatliche Einrichtungen auf Bundes- oder Landesebene. Akteure auf kommunaler Ebene wurden ausgeschlossen	**40**
*Bundesoberbehörden*	Bundesoberbehörden, welche Gesundheit in Deutschland als Schwerpunkt auf der Website aufgeführt oder eine Abteilung/ein Referat zu Public Health im Organigramm aufgelistet haben	12
*Bundesministerien*	Bundesministerien, welche Gesundheit in Deutschland als Schwerpunkt aufgeführt oder eine Abteilung/ein Referat zu Public Health im Organigramm aufgelistet haben	10
*Einrichtungen der staatlichen Aus- und Weiterbildung*	Öffentlich-rechtliche Institutionen mit einem Fokus auf Aus‑, Fort- und Weiterbildung im Bereich Public Health. Oftmals mit zusätzlicher angewandter Forschung im jeweiligen Sektor	4
*Landesoberbehörden*	Oberbehörden auf Landesebene, welche Gesundheit als Schwerpunkt aufgeführt oder eine Abteilung/ein Referat zu Public Health im Organigramm aufgelistet haben	4
*Bundesanstalten*	Bundesanstalten, welche Gesundheit in Deutschland als Schwerpunkt aufgeführt oder eine Abteilung/ein Referat zu Public Health im Organigramm aufgelistet haben	3
*Landesministerien*	Ministerien auf Landesebene, welche Gesundheit als Schwerpunkt aufgeführt oder eine Abteilung/ein Referat zu Public Health im Organigramm aufgelistet haben	2
*Weitere (vorwiegend) staatliche Akteure*	Staatliche Akteure mit Public-Health-Arbeitsschwerpunkt, die keiner anderen Subkategorie eindeutig zuzuordnen waren, da sie bspw. auf Landesebene agieren, aber kein(e) Landesministerium/-oberbehörde darstellen. Hierunter fallen Zusammenschlüsse aus staatlichen Akteuren, auch wenn diese als Verein (ohne staatliche Zuschüsse) organisiert sind	5
**Berufsverbände und Interessenvertretungen**	Berufsverbände oder Interessenvertretungen im Gesundheitsbereich, die berufsständische Interessen der jeweiligen Mitglieder vertreten	**37**
**Wissenschaftliche und akademische Akteure**	Akteure, die im wissenschaftlichen und/oder akademischen Kontext arbeiten und nicht explizit der Privatwirtschaft oder staatlichen Akteuren (öffentlich-rechtlicher Weiterbildung) zugeordnet werden konnten	**31**
*Außeruniversitäre Forschungseinrichtungen*	Öffentliche Institute und weitere Einrichtungen, die nicht im universitären Kontext angesiedelt sind und primär Forschungsarbeit leisten	18
*Wissenschaftliche Fachgesellschaften*	Wissenschaftliche Fachgesellschaften, die sich einem bestimmten Fachgebiet widmen	6
*Lehre und Forschung an Hochschulen*	Universitäten und weitere Hochschulen (Fachhochschulen, private Hochschulen …), die Lehre und Forschung zu Public Health betreiben	3
*Weitere Akteure in der Wissenschaft*	Akademische oder wissenschaftliche Akteure, die keiner anderen Subkategorie eindeutig zuzuordnen waren. Privatwirtschaftliche Forschungsinstitute sind unter Privatwirtschaft aufgeführt	4
**Privatwirtschaftliche Akteure**	Akteure im Gesundheitssektor, die eindeutig gewinnorientiert aufgestellt sind und keiner anderen Kategorie zuzuordnen sind	**22**
*Dienstleistungs- und Beratungsunternehmen*	Unternehmen im Gesundheitssektor, die durch die Bereitstellung immaterieller Güter (z. B. Versicherungen, Infrastruktur, Auftragsforschung) gewinnorientiert arbeiten	13
*Verlage und Presseagenturen*	Verlage, die (medizinische/Gesundheits‑/Public-Health‑)Fachzeitschriften, Bücher oder andere Schriften gewinnorientiert publizieren, sowie Presseagenturen mit Schwerpunkt auf der deutschen Gesundheitspolitik	8
*Hersteller von Arzneimitteln und Medizinprodukten*	Unternehmen, die Arzneimittel und/oder Medizinprodukte herstellen	1
**Internationale Akteure**	Trans- und supranationale Akteure mit Relevanz für Public Health in Deutschland	**21**
*EU-Institutionen*	Akteure der Europäischen Union	7
*UN-Organisationen*	Akteure der Vereinten Nationen (United Nations, UN)	6
*Internationale Fachverbände*	Zusammenschlüsse von mehreren Mitgliedern (Einzelpersonen oder Organisationen), die zu einem spezifischen fachlichen Thema auf internationaler Ebene mit Bezug oder Einfluss auf Public Health in Deutschland arbeiten	4
*Zivilgesellschaftliche internationale Organisationen*	Zivilgesellschaftliche Akteure mit Public-Health-Arbeitsschwerpunkt, die nicht der Subkategorie internationale Fachverbände zuzuordnen waren und die einen Bezug/Einfluss auf Public Health in Deutschland und/oder die Möglichkeit zur Mitgliedschaft für Public-Health-Akteure in Deutschland aufweisen	2
*Weitere internationale Akteure*	Trans- und supranationale Akteure mit Public-Health-Arbeitsschwerpunkt, die keiner anderen Subkategorie eindeutig zuzuordnen waren	2
**Sozialversicherungen**	Träger der gesetzlichen Sozialversicherungen. Privatwirtschaftliche Sozialversicherungsträger wurden der Privatwirtschaft zugeordnet	**17**
*Gesetzliche Kranken- und Pflegeversicherung (GKV & PV)*	Trägerorganisationen der gesetzlichen Kranken- und Pflegeversicherung mit Maßnahmen zum Erhalt und der Wiederherstellung von Gesundheit sowie Leistungen im Falle von Pflegebedürftigkeit	9
*Arbeitslosenversicherung (ALV)*	Sozialversicherung, die u. a. Lohnersatzleistungen, Maßnahmen zum Arbeitsplatzerhalt und zur Arbeitsvermittlung leistet. Trägerin ist die Bundesagentur für Arbeit	1
*Gesetzliche Rentenversicherung (GRV)*	Sozialversicherung, die u. a. finanzielle Leistung im Alter, Rente bei Erwerbsminderung und Maßnahmen zur Wiederherstellung der Arbeitsfähigkeit umfasst	1
*Gesetzliche Unfallversicherung (GUV)*	Sozialversicherung, die Maßnahmen zur Verhütung von Arbeitsunfällen und Berufskrankheiten umfasst sowie Leistungen bei deren Eintritt	1
*Weitere Akteure im Bereich Sozialversicherungen*	Akteure der Sozialversicherungen, die keiner (einzelnen) anderen Subkategorie zuzuordnen waren	5
**Stiftungen**	Akteure mit Public-Health-Arbeitsschwerpunkt, die entweder ihrer Rechtsform nach eine Stiftung sind oder sich selbst als Stiftung bezeichnen. Dazu können auch solche Institutionen gehören, die ihrer Rechtsform nach eingetragene Vereine oder eine gemeinnützige GmbH (gGmbH) sind	**13**
**Akteure der Selbstverwaltung im Gesundheitswesen**	Träger des Gesundheitswesens, die sich selbst organisieren und verwalten. Hierzu gehören die Mitglieder des gemeinsamen Bundesausschusses (G-BA) und weitere berufsständische Akteure der Selbstverwaltung (z. B. Kammern)	**11**
**Projektträger**	Institutionen, die durch finanzielle Förderung und Verwaltungsdienstleistungen die Durchführung von Gesundheitsprojekten unterstützen	**4**
**Unabhängige Sachverständigenräte**	Auf Rechtsgrundlage einberufene Gremien, die unabhängige Gutachten zu medizinischen/gesundheitlichen Fragestellungen entwickeln und damit politische Entscheidungsträger:innen informieren	**2**

## Ergebnisse

**Figure 1:**
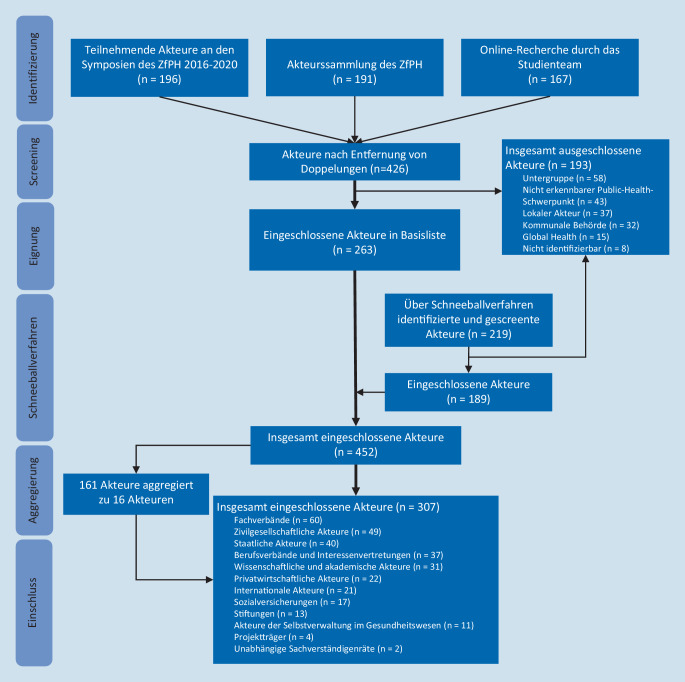

Insgesamt wurden 645 Akteure identifiziert und gescreent. Von diesen wurden 452 Akteure (70,1 %) eingeschlossen. 263 Akteure (58,2 %) wurden im initialen Screening und 189 Akteure (41,8 %) im Rahmen des Schneeballverfahrens eingeschlossen. Von den 452 eingeschlossenen Akteuren wurden 161 (35,6 %) strukturell ähnliche Akteure zu 16 aggregierten Akteuren zusammengefasst. Die Akteursübersicht umfasst somit insgesamt 307 Akteure (s. zusätzliches Onlinematerial). Das mehrstufige Verfahren der Identifikation von Akteuren, Screening, Aggregierung und Kategorienzuordnung ist im Flussdiagramm abgebildet (Abb. 1). Die Anzahl ausgeschlossener Akteure pro definiertem Ausschlusskriterium ist in Tab. 1 dargestellt.

Es wurden insgesamt 12 Ober- sowie 30 Subkategorien induktiv gebildet. Die Größe der Kategorien, gemessen an den enthaltenen Akteuren, und die Anzahl der Subkategorien variiert deutlich zwischen den Kategorien. Die höchste Anzahl an Akteuren weisen die Oberkategorien Fachverbände (*n* = 60; 19,5 %), zivilgesellschaftliche Akteure (*n* = 49; 16,0 %) und staatliche Akteure (*n* = 40; 13,0 %) auf (Tab. 2).

**Figure 2:**
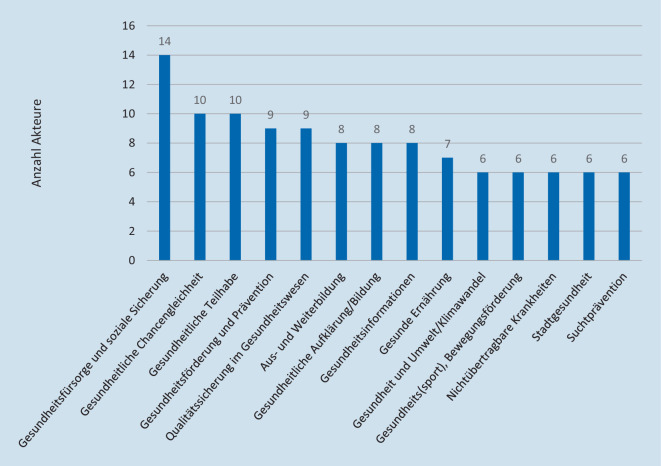

Für einen Großteil der Akteure (*n* = 180) in den 4 Oberkategorien Fachverbände, zivilgesellschaftliche, wissenschaftliche und akademische sowie staatliche Akteure konnte die Zuordnung zu einem primären inhaltlichen Themenschwerpunkt vorgenommen werden, während eine solche thematische Eingrenzung in den übrigen Oberkategorien aufgrund des institutionellen Charakters (z. B. Organe der Selbstverwaltung) oder eines sehr breiten Arbeitsspektrums in den meisten Fällen nicht möglich war (*n* = 22 Akteure mit diversen Themenschwerpunkten). Die 14 am häufigsten identifizierten Einzelthemenschwerpunkte der 180 Akteure werden in Abb. 2 dargestellt.

**Figure 3:**
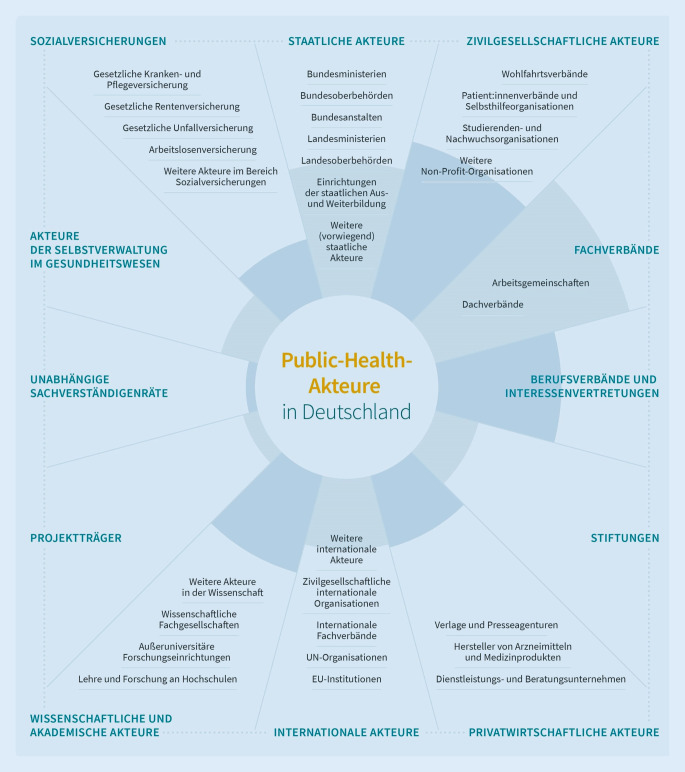

Zusätzlich zu der tabellarischen Übersicht aller eingeschlossenen Akteure wurden eine Infografik des Kategoriensystems (Abb. 3) sowie eine interaktive Visualisierung der Akteursübersicht erstellt, die öffentlich auf der Homepage des NÖG zugänglich ist (www.noeg.org).

## Diskussion

Diese Arbeit bietet eine Zusammenstellung der Akteursgruppen, die in Public Health in Deutschland tätig sind, sowie eine detaillierte Auflistung und Kategorisierung von insgesamt 307 überregional agierenden Institutionen. Die Akteursübersicht und die identifizierten Themenschwerpunkte zeigen die Breite und thematische Vielfalt von Public Health in staatlichen, zivilgesellschaftlichen, akademischen und privatwirtschaftlichen Strukturen in Deutschland. Dabei weisen die Akteure nicht nur ein breites inhaltliches Spektrum, sondern auch eine große Varianz in der Ausprägung ihrer Schwerpunktsetzung auf: Während einige Akteure einen reinen Public-Health-Schwerpunkt aufweisen, stellt bei anderen Akteuren öffentliche Gesundheit nur ein Themenfeld von vielen dar. Unsere Arbeit bietet damit einen wichtigen Baustein zu einem besseren Verständnis von öffentlicher Gesundheit in Deutschland als komplexes Public-Health-System.

### Die Ergebnisse im Kontext der Forschung zu Public-Health-Systemen

Wir kategorisierten die Public-Health-Landschaft anhand institutioneller Akteure, ähnlich den Übersichten institutioneller Akteure des ÖGD und des Gesundheitssystems in Deutschland [14, 15]. Public-Health-Systeme können jedoch auf verschiedene Arten definiert werden. Neben der Beschreibung von staatlichen und nichtstaatlichen Akteuren, die „gemeinsam Verantwortung für physische und soziale Umgebungen der Bevölkerung tragen“ (übersetzt nach Jarvis (2020) [22]), sind auch Beschreibungen von Public-Health-Systemen anhand der Kernfunktionen oder Aktivitäten in diesen Systemen möglich [22]. So bilden beispielsweise die *Essential Public Health Operations* (EPHOs) die Möglichkeit einer Definition von Kernfunktionen in Public Health, welche von der Weltgesundheitsorganisation (WHO) für die Region Europa als Bestandteil einer Evaluation von Public-Health-Diensten vorgeschlagen wurde [23].

Auch in anderen Ländern wie den USA und Israel wurden ähnliche Kerndienste, beziehungsweise -funktionen, für die staatlichen Public-Health-Systeme definiert [24] oder, wie im Jahr 2007 in England, Strukturen, Kapazitäten und Veränderungen des Systems analysiert [25]. Detaillierte Beschreibungen von lokalen Public-Health-Systemen sind mit Methoden der Netzwerkanalyse möglich und können eine wertvolle Ressource für lokale Akteure darstellen [18]. Jarvis et al. zeigen in ihrer systematischen Übersichtsarbeit weitere Konzeptualisierungen von Public-Health-Systemen und ihre Positionierung in Gesundheitssystemen [22]. Damit stellt die vorliegende Übersicht der Akteursgruppen im deutschen Public-Health-System eine von mehreren Möglichkeiten zur Kategorisierung dieses Systems dar.

### Implikationen für Forschung und Praxis

Die Übersicht institutioneller Akteure ermöglicht vielfältige Nutzungsformen für Anwender:innen. Sie kann die berufliche Orientierung von Public-Health-(Nachwuchs‑)Fachkräften erleichtern und trägt dazu bei, eine wichtige Lücke zu füllen, da die Vielfalt der Berufswege in Public Health in Deutschland bislang oftmals unbekannt oder intransparent war [13, 26]. Für Akteure in und außerhalb des Public-Health-Systems fördern die vorliegenden Ergebnisse Möglichkeiten zur Vernetzung und Kollaboration. Die zunehmende institutionelle Zusammenarbeit von Public-Health-Akteuren in Deutschland im ZfPH, in der Deutschen Gesellschaft für Public Health, in der Deutschen Gesellschaft für Sozialmedizin und Prävention, im NÖG sowie in Initiativen wie dem *Kompetenznetz Public Health COVID-19 *(www.public-health-covid19.de) zeigt den Bedarf und das Interesse an Vernetzung, Austausch und Kooperation in der Fachgemeinschaft. Eine besondere Rolle kann die Übersicht der Akteure für den Austausch von Wissenschaft und Praxis oder Politik bieten: So geht strategische Wissenstranslation mit einer systematischen Identifikation relevanter Stakeholder einher [27–29], bei der unsere Übersicht eine wertvolle Ressource darstellen kann. Und schließlich kann die Übersicht der Public-Health-Akteure in Zusammenschau mit bestehenden Übersichten zum ÖGD und Gesundheitssystem [14, 15] ein tiefergehendes Verständnis der aktuellen Public-Health-Landschaft in Deutschland vermitteln, was etwa für die Unterstützung der Reformprozesse in der öffentlichen Gesundheit in Deutschland im Rahmen einer nationalen Public-Health-Strategie [11] von Bedeutung ist.

Die vorliegende Akteursübersicht erhebt dabei keinen Anspruch auf Vollständigkeit, vielmehr ergeben sich hierauf aufbauend Möglichkeiten für ergänzende Forschungsprojekte. So sind Erweiterungen der Übersicht mit einem HiAP-Fokus denkbar: Der HiAP-Ansatz nimmt die gesundheitlichen Auswirkungen in allen Politikbereichen im Sinne der Ottawa-Charta in den Blick und erfordert eine umfassende intersektorale Zusammenarbeit mit Akteuren außerhalb des Gesundheitssektors [30, 31]. Die multisektorale gesundheitsbezogene Zusammenarbeit in der SARS-CoV-2-Pandemie bietet Chancen für HiAP in Deutschland [7], sodass Kartierungen der HiAP-Akteure zu definierten Fragestellungen sinnvoll erscheinen.

Die vorgestellte Übersicht kann auch als Ausgangspunkt für tiefergehende Analysen der Akteursbeziehungen oder eine Kategorisierung anhand von spezifischen inhaltlichen Themenschwerpunkten der Akteure (z. B. Klimawandel, Ernährung, Stadtgesundheit) oder anhand der EPHOs dienen. Zudem können zukünftige Forschungsprojekte die vielseitigen Ausbildungs- und Berufswege in der deutschen Public-Health-Landschaft noch weitergehend im Detail systematisieren und aufzeigen. Ebenso können Ergänzungen der Übersicht um eine breitere Vielfalt internationaler Akteure hilfreich sein, um die globalen Verbindungen und Einflüsse des Public-Health-Systems aufzuzeigen. Für diese Ansätze spielen neben der Dokumentenanalyse weitere Methoden, wie beispielsweise *Key Informant Interviews* (Interviews mit Schlüsselpersonen) und Netzwerkanalysen, eine Rolle.

### Stärken und Limitationen

In dieser Arbeit sind wir wie viele andere auch zunächst der Herausforderung begegnet, Public Health so zu definieren, dass eine Operationalisierung möglich ist [22, 25]. Daraus ergeben sich zum Teil Unschärfen und mögliche Lücken. So bedingt die angewandte Definition von Public-Health-Akteuren den Ausschluss von Organisationen und Institutionen, die zwar direkt oder indirekt die öffentliche Gesundheit beeinflussen, dies aber nicht als eigentlichen Arbeitsschwerpunkt haben. Die Zuordnung der Akteure spiegelt ihre Kernbereiche wider, bildet jedoch nur begrenzt das individuelle Arbeitsspektrum der Akteure ab. Auch wäre bei einer Vielzahl von Akteuren eine Zuordnung zu mehreren Ober- und Subkategorien möglich gewesen, auf die wir jedoch aus Gründen der Übersichtlichkeit verzichtet haben.

Die kommunale Ebene, insbesondere Gesundheitsämter und angrenzende Verwaltungsbereiche, die Breite an Lokalgruppen, Initiativen, Netzwerken und die dort aktiven Individuen sind für die Förderung und den Schutz öffentlicher Gesundheit vor Ort unverzichtbar. Diese Ebene konnte – gerade wegen ihrer Vielzahl an Akteuren und dem Ziel eines deutschlandweiten orientierenden Überblicks – in dieser Arbeit nicht dargestellt werden. Eine systematische Erfassung der kommunalen Ebene, beispielsweise auch die detaillierte Kartierung der Public-Health-Landschaft in einzelnen Städten und ländlichen Regionen, kann ein wichtiger Schritt sein, um Transparenz und Zusammenarbeit auf kommunaler Ebene zu fördern sowie Bedarfe und bestehende Angebote zu identifizieren.

Unter Berücksichtigung dieser Limitationen weist die Methodik unserer Arbeit entscheidende Stärken auf: Unsere Arbeit stützt sich auf eine umfangreiche Übersicht von Public-Health-Akteuren, die zwischen 2016–2020 das ZfPH als eine der größten Public-Health-Veranstaltungen in Deutschland besucht hatten oder hierzu angemeldet waren, sowie auf eine weitere Akteurssammlung durch die Geschäftsstelle des ZfPH. Mit der ergänzenden Onlinerecherche und einer erweiterten Suche im Schneeballverfahren sind wir zuversichtlich, eine Vielzahl relevanter Institutionen und Organisationen identifiziert zu haben.

### Fazit

Dies ist unserem Wissen nach die erste umfassende Übersicht überregionaler institutioneller Public-Health-Akteure in Deutschland. Durch die gewählte Methodik konnte ein aktueller Querschnitt einer Vielzahl an Public-Health-Akteuren erfasst und induktiv in einem Kategoriensystem angeordnet werden. Um zukünftigen Veränderungen in der Public-Health-Landschaft gerecht zu werden und eine dynamische Erweiterung dieser Arbeit zu ermöglichen, steht eine interaktive Visualisierung der Akteursübersicht für Ergänzungen zur Verfügung (www.noeg.org).

Diese sowie weitere Forschungsarbeiten zu den inhaltlichen Arbeitsschwerpunkten, Funktionen oder den Strukturen von Public-Health-Akteuren können Nachwuchsfachkräften die Vielfalt des Berufsfeldes transparent aufzeigen, zu Zusammenarbeit und Vernetzung zwischen Akteuren beitragen und sind auch vor dem Hintergrund bevorstehender Reformen des öffentlichen Gesundheitswesens in Deutschland von Bedeutung.

## Supplementary Information




